# Lower Airway Virology in Health and Disease—From Invaders to Symbionts

**DOI:** 10.3390/medicina54050072

**Published:** 2018-10-13

**Authors:** Lina Jankauskaitė, Valdonė Misevičienė, Laimutė Vaidelienė, Rimantas Kėvalas

**Affiliations:** Department of Paediatrics, Medical Academy, Lithuanian University of Health Sciences, LT-50161 Kaunas, Lithuania; valdonemis@yahoo.com (V.M.); laimavaidel@icloud.com (L.V.); rimantas.kevalas@kaunoklinikos.lt (R.K.)

**Keywords:** chronic airway disease, respiratory virome, bacteriophages, antiviral immunity, host–virus interaction, treatment

## Abstract

Studies of human airway virome are relatively recent and still very limited. Culture-independent microbial techniques showed growing evidence of numerous viral communities in the respiratory microbial ecosystem. The significance of different acute respiratory viruses is already known in the pathogenesis of chronic conditions, such as asthma, cystic fibrosis (CF), or chronic obstructive lung disease (COPD), and their exacerbations. Viral pathogens, such as influenza, metapneumovirus, parainfluenza, respiratory syncytial virus, or rhinovirus, have been associated with impaired immune response, acute exacerbations, and decrease in lung function in chronic lung diseases. However, more data have attributed a role to Herpes family viruses or the newly identified *Anelloviridae* family of viruses in chronic diseases, such as asthma, idiopathic pulmonary fibrosis (IPF), or CF. Impaired antiviral immunity, bacterial colonization, or used medication, such as glucocorticoids or antibiotics, contribute to the imbalance of airway microbiome and may shape the local viral ecosystem. A specific part of virome, bacteriophages, frames lung microbial communities through direct contact with its host, the specific bacteria known as *Pseudomonas aeruginosa* or their biofilm formation. Moreover, antibiotic resistance is induced through phages via horizontal transfer and leads to more severe exacerbations of chronic airway conditions. Morbidity and mortality of asthma, COPD, CF, and IPF remains high, despite an increased understanding and knowledge about the impact of respiratory virome in the pathogenesis of these conditions. Thus, more studies focus on new prophylactic methods or therapeutic agents directed toward viral–host interaction, microbial metabolic function, or lung microbial composition rearrangement.

## 1. Introduction

The human virome is a versatile component of a microbial ecosystem. The human virome consists of eukaryotic viruses (viruses targeting and infecting eukaryotic cells), bacterial virome (bacteriophages infecting human-hosted specific bacteria) and a group of virus-derived genetic elements integrated into host chromosomes, such as human endogenous retroviruses, endogenous viral elements, or prophages, which represent “footprints” of previous viral infections [1]. For a long time, eukaryotic respiratory viruses were thought to be the most common cause of acute airway infections and their role was evident in acute exacerbations of asthma, chronic obstructive pulmonary disease (COPD), cystic fibrosis (CF), or idiopathic pulmonary fibrosis (IPF). Improvement in culture-independent microbiological techniques enabled broader analysis of previously known viruses and discovery of new local viral communities in the respiratory tract [2,3,4,5,6,7,8,9,10]. The rise of high-throughput sequencing revealed new viral intrinsic constituents in airway microbiomes of healthy individuals and/or asymptomatic cases. This finding contradicted the long-lasting understanding of distal airway sterility. A healthy airway was revealed to sustain numerous viral communities compared to the chronic inflammatory state. Local bacteriophage or eukaryotic viruses in the distal airway contribute to chronic disease pathogenesis, influence the human immune system, and govern the health–disease balance. In addition, bacteriophages, forming the majority of the virome in a healthy lung, are attributed a crucial role to impact human lung immunity via its bacterial host. Moreover, transitory respiratory viruses have been suggested to portray an essential role in the pathogenesis of chronic lung disease exacerbations. New studies assigned more immune-modulatory functions to the human lung virome in chronic respiratory inflammatory conditions, such as CF, asthma, COPD, or IPF. Morbidity and mortality of chronic respiratory tract diseases, however, remains very high. Therefore, new therapeutic agents directed towards microbial metabolic function, lung microbial composition rearrangement, or viral–host interaction are attracting increasing interest. In this review, we will focus on the current state of the distal airway virome and its role in physiology and pathophysiology of chronic lung conditions. We will discuss the importance of the pulmonary virome in chronic inflammation, host immunity, and virus–host interaction. Moreover, we will address the possibility of new prevention and treatment options.

## 2. Lungs Are Not Sterile

For a long time, it was thought that the lung is a sterile organ. Any sort of microbial detection in human sputum, bronchoalveolar lavage fluid (BALF), or postmortem lungs was considered to be an acute or exacerbated airway infection or interpreted as probable sample contamination from the nasopharynx [11]. Recently, several studies have concentrated on airway microorganisms and demonstrated that the regular lung microbiome is without any inflammatory or infectious background [12,13]. A healthy lower respiratory tract (LRT) bacterial and viral microbiome exchanges greater membership with the microbial elements in the mouth than with those detected in air [14]. The richness of this microbiome is observed to decline in the healthy LRT at a greater distance from the larynx. Anatomical peculiarities of the bronchial tree appear to play a significant role in microbial distribution [15]. The surface of the respiratory tract is covered by a protective layer of mucus. This mucosal shield provides a genuine portal for a substantial population of microbial symbionts, including different viral communities. Unique microorganisms are not only harbored in airway mucosa, but can also be found in BALF, especially in severe cases of chronic lung disease [16]. As follows, lower airway mucosa and secretions can be divided into two different distinct niches of microbial growth. These findings were first confirmed and attributed exclusively to human gut, but may be a part of two different subunits of the respiratory system. Likewise, transitory viral pathogens (e.g., influenza, parainfluenza, rhinovirus (RV), respiratory syncytial virus (RSV), or adenovirus) can temporarily supplement the virome of the airway [17,18]. A healthy LRT microbiome is characterized by extensive inter-individual biodiversity. By contrast, microbial changes under specific disease states could be labelled a diagnostic hallmark and determine an acute condition or predict exacerbation of a chronic one. Compared to other environmental viromes, the respiratory tract virome is less rich in species. The natural barriers, including physical and both innate and adaptive immunity, may result in low viral diversity. Healthy airways have been shown to be populated by phages and DNA viruses. Eukaryotic viral communities in a healthy state may represent transient infectious agents rapidly cleared by immune system cells. By contrast, these viruses could mirror persistent infection in a chronic airway disease state [8]. In addition, novel viral agents, such as the *Anelloviridae* family of viruses, have been identified in healthy human lung, stable chronic disease conditions, and blood samples [3,10,14,15,19]. Early infection with Anelloviruses may be common in infancy and viral loads are decreasing over time [19]. However, the pathogenic role of these viruses and their persistence is not fully understood. More recent data have shown that the *Anelloviridae* family, such as Torque-Teno viruses (TTVs), may be associated with fever or exacerbated chronic lung disease. More moderate Anellovirus loads were detected in patients suffering graft rejection [2,3,10,19]. The role of TTVs in host–viral interaction is still debatable. However, increased TTV loads are associated with lower CD3+ and CD4+ T cell numbers, a greater B cell count, and eosinophil activation in circulation, revealing the immunomodulatory activity of TTVs [20]. Viral metabolic profiles are distinct in health and disease states, and such changes in metabolic function can be considered in differentiating a healthy or stable condition from exacerbation [8].

Respiratory virome components can be arranged into two major categories: Commensals and opportunistic pathogens. The equilibrium between being a commensal or becoming a pathogen is determined by many diverse direct and indirect factors of the host and viral community itself. The welfare of a microbial ecosystem in health and disease conditions depends on such factors as microbe immigration, microbial elimination, and microbiome reproduction rates. It is directly related to the local growth conditions and environmental factors in the airway, as well [21]. The lung virome is not an exception. Changes in local bacterial populations during different acute or chronic diseases can influence or vice versa be influenced by transitory or resident viral inhabitants in the human airway [1,17,18,22]. Viruses may compete with each other choosing a two-way action—either to protect their environment from possible invaders (other viral pathogens or bacterial threat) or to overthrow the native resident viral communities. Published data do show the crucial role of viral inhabitants in the LRT in modulating and priming host immunity. A low-level immune response is continuously stimulated by different transitory viruses in asymptomatic individuals. Increased loads of viruses (e.g., RSV or adenovirus) were detected in stable chronic airway disease patients. This may contribute to pathogenesis of persistent conditions and may provoke host immune response [17]. Several studies displayed the same viral load enhanced inflammatory response in chronic conditions compared to control subjects [23]. Increases in chemokines during clinically stable COPD recruited different inflammatory cells, such as neutrophils, macrophages, or T-cells. These pro-inflammatory cells were not only found in greater numbers, but they also presented different phenotypes compared to those of controls [23]. Chronic respiratory diseases, such as asthma or COPD, have been associated with impaired barrier function of the airway, which could explain the different response to the same pathogen or same viral load, compared to healthy subjects.

In addition, the essential role of viral species should be attributed to the control of other pathogens within the respiratory tract. A substantial focus must be concentrated on human-hosted airway bacteriophages. Various bacterial and eukaryotic viral residents have been of interest in the human airway, as they may directly cause morbidity and mortality of the macroorganism. However, the phage inhabitants have been ignored for a long time. These components of the respiratory virome have the potential to shape and indirectly influence disease state or human immune response by regulating their bacterial host [20,21,22,23,24,25,26,27].

## 3. Human Lung—A “Pool” of Bacteriophages

Bacteriophages are obligate parasites and their survival and fitness directly depend on their bacterial host. Phages must also manipulate gene expression of their host cells to evade possible superinfection with other phages.

Various studies have shown that bacteriophages serve as potential virulence gene reservoirs. Through the process, bacteriophages can be used as vehicles and mediate the transfer of different virulence genes into a bacterial host [20,25,28,29]. They also influence virulence genes to travel between bacterial species on prophage induction [30]. Most bacterial virulence factors, such as bacterial fitness, colonization, adhesion, invasion, or toxins, are encoded by various bacteriophages. In addition, approximately 20% of bacterial genetic content is acquired [29]. To note, certain bacteria can use these phage-encoded antimicrobial resistance genes to enhance their own survival by inhibiting other bacterial pathogens [22,29]. Respiratory tract microbiota is commonly exposed to various antimicrobials, consequently forming a local antimicrobial resistance gene pool. In chronic conditions, such as CF, phages may maintain a reservoir of antimicrobial resistance genes [22,25]. These genes can be mobilized and disseminated. Together with environmental resistomes and gut resistomes, this leads to complex genomic assembly in a prokaryotic viral population. This selective pressure, especially high in chronic lung diseases, stimulates microbial evolution and antibiotic resistance [21,22], and is due to bacterial competition for resources [25]. Of note, it was shown that administration of antibiotics induces phage and phage-mediated lateral gene transfer [30]. Phage communities differ in their metabolic profiles between healthy individuals and disease and chronic or acute respiratory tract infections [2,8,21,25]. A dataset of CF samples was enriched with the two top-ranking metabolic pathways: Carbohydrates and amino acids and derivates. In addition, the core metabolic profile of the CF phage included virulence and cell wall-related functions. Functions associated with synthesis of the biofilm structural component rhamnose or iron uptake were also detected. This finding reflects their tremendous adaptation to a specific environment, environmental changes over time, and both a very distinct relationship with a bacterial host in the respiratory tract and its genome adjustment to a resident niche [8,21]. It is feasible that certain bacterial pathogens exist in a less virulent state and prophages are induced under specific stimuli. Reactive oxygen species (ROS) generated and released by leucocytes and antimicrobials or H_2_O_2_ produced by neutrophils induce prophage stimulation [20,21,31]. The association between bacterial host and prophage can induce these elements to produce phage particles. These bacteria-associated particles may prime innate and adaptive immune responses. Accordingly, the human immune system can “spot” bacteriophages and, in another manner, phages can be engulfed by dendritic cells (DCs), and their antigens may be further presented to T-cells. Such preconditioning of cellular-immunity results in cytokine production [24]. Distinctly, phages can destroy local bacterial populations and reinforce overgrowth of other more pathogenic bacteria. As shown in a few studies, bacteriophages are not always involved in pathogenic processes due to their extraordinary flexibility and abilities to adapt in local environments. It was observed that CF and healthy individuals may share very similar respiratory tract viromes due to their similar living settings [8]. These findings confirm that the villain–pacifier balance depends not only on the microorganism alone, but also on the host immune system and its interaction, with a probable viral threat of a high significance.

## 4. Cystic Fibrosis

Cystic fibrosis (CF) is the most common fatal autosomal recessive genetic disorder in the Caucasian population. The dysfunction of the cystic fibrosis transmembrane regulator protein (CFTR) leads to abnormal chloride and sodium transport across the pulmonary epithelium. As a result, a polymicrobial community inhabits CF airways and lung function progressively declines. Increased production of pro-inflammatory cytokines or a deficient antiviral innate immune response may be relevant to CF. The inflammatory response is virus- or even viral group-dependent. The impaired innate immune response decreases viral clearance and boosts viral replication [32]. Upregulation of nuclear factor kappa B (NFκB) and endoplasmic reticulum (ER) stress-induced by accumulation of CFTR in the ER is linked to baseline inflammation in lungs with CF. Impaired signaling pathways for nitric oxide (NO) and reduced production of nitric oxide synthetase 2 (NOS2), which is crucial to defend against viruses (e.g., rhinovirus, RSV, or influenza virus), or oligoadenylate synthetase 1 (OAS1), which contributes to viral degradation of double-strand (ds) RNA, are observed in CF epithelium [32].

The pediatric CF microbiome has been shown to be more assorted compared to that of adults. However, it seems that the airway microbiome undergoes strict strain selection, which is exacerbation-related. This diversity is lost, leading to several predominant pathogens. Recent culture-independent studies have shown the role of antibiotics in changes of the CF microbiome. It seems that antibiotic therapy does not target the dominant pathogen. Moreover, repeated use may affect healthy airway commensals [33]. In addition, several studies uncovered previously underdiagnosed microbial species “supporting” CF exacerbation. These species contribute to the processes of inflammation and airway destruction [7,8,34].

With a metagenomic approach, several human herpesviruses (HHVs), including the Epstein–Barr virus (EBV), were detected in CF lungs. EBV infection in adolescent CF patients has been linked to exacerbations of respiratory conditions and poor outcomes [8]. CF children infected with the varicella zoster virus (VZV) show increased morbidity, more clinical complications, and exacerbations [32]. Other DNA viruses, such as TTVs and retroviruses, were associated with persistent infection, exacerbations, and poor clinical outcome in CF patients [7,35]. DNA viruses and common respiratory RNA viruses (e.g., RSV, RV, or influenza virus) have been linked to more severe clinical illness and associated with deterioration in lung function. Studies using PCR analysis have reported respiratory virus detection rates between 50% and 60% in CF patients. At least one respiratory virus was identified in 60% of children presenting with CF exacerbation [36]. Rhinoviruses do persist longer in CF patients compared to healthy individuals. Increased RV loads correlate with impaired pulmonary function and decreased antiviral immunity. Viral infections with RSV, RV, influenza virus, parainfluenza virus, or adenovirus were also associated with an acute increase in CFU/mL of *Pseudomonas aeruginosa* in CF airway samples. In vitro data have shown that RV infection can liberate *P. aeruginosa* from biofilms. These planktonic bacteria were more pro-inflammatory in vitro [37]. A study by Ramirez and colleagues demonstrated an increased expression of many pro-inflammatory cytokines and signaling intermediate genes associated with influenza virus-induced CF exacerbations. Moreover, the influenza virus increased expression of tumor necrosis factor alpha (TNF-a) and its downstream effectors TNF receptor associated factor (TRAF) 3 and TRAF6, meanwhile, RV induced chemokines in mononuclear cells [38]. Additionally, interferon (IFN) signaling is impaired in CF airways. Prior infection with *P. aeruginosa* has been proved to suppress IFN responses to subsequent viral infections in CF lungs [39]. In addition, viral coinfections are quite common in CF patients. At least two viruses are detected in these patients (e.g., RV is associated with human metapneumovirus (hMPV), influenza virus type A, adenovirus, or parainfluenza virus). This infection contributes to hospital admission rates and increases pathogenicity. CF children are most affected by these coinfections [32]. It is important to mention a strong correlation between viral infection and a rise of colonization with *P. aeruginosa*. Increased levels of Pseudomonas antibody before colonization with this pathogen was found in CF patients with RSV-induced infection by Peterson et al. [40]. More studies demonstrated that RSV can behave as a coupling agent between some strains of Pseudomonas and airway epithelial cells [41].

The essential feature of a CF lung is its own bacteriophage community, which directly embodies the persistence of bacterial species. Intensive antibiotic therapies have extended the life expectancy of CF patients. The role of antibiotic use in CF and well-known phage transduction mechanisms can explain the high antibiotic resistance rates in these patients. Transduction is one of the most potent tools of gene dissemination that allows bacteria to become more pathogenic and evolve resistance to different antimicrobials. Many genetic exchanges occur by horizontal gene transfer (HGT) due to antimicrobial treatment and selective pressure. This leads to bacterial selection and multidrug-resistant pathogen evolution. Diverse phages are associated with bacterial agents, such as *P. aeruginosa*, *Staphylococcus aureus*, or *Burkholderia cenocepacia*, and contribute to bacterial adaptation within the respiratory tract niche in a CF lung [7,22]. These bacteriophages do mobilize antibiotic resistance genes in the CF sputum microbiota. Pathogenic bacteria “benefit” from phage-encoded antibiotic resistance genes, cause inhibition of growth of other strains, and enhance their own survival in CF lungs [2]. Bacteriophages facilitate biofilm formation and adaptation within the host [8,28,42]. Phages have been attributed a role in modulating phenotype differences in *P. aeruginosa* colonies and associated biofilms. This leads to highly virulent colony variants [21,25]. *P. aeruginosa* implicates aromatic acids in carbon sources, as regulators of quinolone signaling and biofilm formation in CF sputum. Phages do present with anaerobic aromatic catabolism genes. Aromatics can be degraded via bacteriophages to reduce the exopolysaccharide layer, allowing penetration of the biofilm [8]. In addition, microbes in CF lungs are subjected to nutrient limitations and can escape phage predation [21]. Bacteriophage communities were found to be common to all CF patients, but different from healthy subjects. The bacteriophage population in the respiratory tract of CF patients was present with a specific metabolic profile, showing an immense adaptation to the distinct character of CF mucus. Phage metabolism reflects bacterial–host metabolism. In addition, gaining phage resistance bacteria may shift their metabolic profiles [21]. Metabolomic studies of CF patients showed enrichment with aromatic amino acids and phosphate. This finding suggests that specific bacteria favor rearrangement in a local microbial niche of the LRT. Such competition based on metabolic environment synchronizes with alterations in the lung virome and may lead to disease exacerbation [24].

## 5. Asthma

Asthma, a heterogeneous syndrome with intermittent wheezing and chronic airway inflammation, is still considered an enigmatic disease. Microbiota of the airway, as well as gut, is attributed one of the most important roles in the etiology of this condition [43]. In addition, distinct microbial components were recovered from the bronchial secretions and mucosa layer [16]. Respiratory syncytial virus, a very common viral agent in childhood, is demonstrated to be a risk factor to increase respiratory morbidity. RSV has been shown to incline asthma/wheezing together with such factors as genetic susceptibility or exposure to environmental tobacco smoke. Impaired innate immunity implies a mechanism for both virus-induced exacerbations and a link between a predisposition to respiratory viral infections in the successive development of asthma. Recently, a principal part of asthma development was assigned to rhinovirus infection. Asthma exacerbations and disease severity are linked to this virus. RV has been isolated in approximately 90% of pediatric cases of asthma exacerbations and has been shown to have a close connection to hospitalization rates [44]. Additionally, RV-related bronchiolitis is associated with greater lung function deficits and could predict persistent wheezing or asthma [45]. A study conducted by Ki-Hyun Seo et al. detected RV in 45.5% of the LRTs of asymptomatic stable asthma patients. Moreover, those infected with rhinovirus are more likely to have more severe LRT infections, with longer-lasting symptoms [46]. Few studies have shown that RV induces expression of major epithelial mucin MUC5AC, leading to mucus hypersecretion. Further, RV affects airway epithelial cells and the secretion of matrix metalloprotease 9 (MMP-9) and vascular–endothelial growth factor (VEGF). Epithelial barrier function can be impaired during allergic airway inflammation. Damaged epithelial cells favor RV replication within the asthmatic airway and drive infection of macrophages. RV-induced interferon responses are decreased [17,47]. The IFN-mediated innate antiviral response is one of the most crucial antiviral immunity systems [48]. Together with the NFκB signaling cascade, it induces hundreds of antiviral and pro-inflammatory genes. Interferon stimulated genes (ISGs), such as Mx proteins, OAS, and viperin, assist as potent antiviral proteins, inhibiting viral replication and hampering viral spread. Thus, IFN deficiency results in more severe and prolonged respiratory symptoms in asthmatic patients. The influenza virus is another common virus in asthmatic patients [46]. During the influenza virus pandemics in 2009, asthma was one of the risk factors associated with increased hospitalization among children and adults. It was observed that children were more prone for pH1N1 infection during pandemics. Moreover, 13% and 43% of children infected with pH1N1 were positive for RSV and RV, respectively. In addition, asthmatics on steroids had increased risk for pH1N1 due to transient immunosuppression. The clear mechanism of the influenza virus and the asthmatic airway is still under investigation [49]. However, a few animal studies have already provided significant data considering the role of the influenza virus in asthma. As previously mentioned, it was shown that the IFN signaling pathway, which is a safeguard from virus-induced cytotoxicity, is impaired in the asthmatic airway. Moreover, transforming growth factor beta (TGF-b) plays a crucial role in the asthmatic lung. This factor inhibits virus-induced pathology [50], promotes IgA production, and is involved in respiratory tissue repair and remodeling [51]. Downregulation of mucosal antibody responses was shown in animal studies by Furuya et al. [50]. Keegan et al. proposed a theory that allergen-associated molecular patterns may stimulate pattern recognition receptors on alternatively activated macrophages (AAMs) [52]. This phenomenon can lead to the immediate production of various interleukins (ILs), such as IL-4 and IL-13, and chemokines activating DCs and other cells. DCs migrate to the draining lymph node and stimulate naive CD4+ T-cells to allergen-specific Th2 or memory cells. Activated Th2 cells further migrate to the site of inflammation in the airway and induce IL production, which promotes AAM differentiation, mucus production, further chemokine production, and eosinophilic infiltration. Together with lung epithelial cells, AAM-produced chemokines recruit more inflammatory cells. Exposure to a viral infection may stimulate memory Th2 cells to produce higher levels of ILs and participate in a positive amplification loop [52]. Most epidemiological studies have focused on the association of RSV and RV with asthma development or pathogenesis. However, human bocavirus (HBoV) has accounted for 10% of bronchiolitis cases [53,54]. One study suggested that HBoV is responsible for one-quarter of recurrent wheezing cases in children [55]. However, those patients were followed up for only one year. The role of human metapneumovirus in asthma risk was demonstrated by Garcia-Garcia et al. This group reported that hMPV accounted for 10% of hospitalized children with bronchiolitis or recurrent wheezing and 40% of asthma diagnoses in hMPV-positive children [56,57]. In addition, a few studies revealed bacterial biofilm formation in severe chronic asthma patients [16]. This could imply the existence of specific phage communities within the asthmatic lung.

The initial trigger of each asthma endotype occurs at the bronchial epithelium. Bronchial epithelial cells are responsible for barrier and immune protection against agents such as viruses. However, it is still not clear if different viruses or alterations in the airway virobiome could predict asthma endotype or suggest clusters of specific phenotypes.

## 6. Chronic Obstructive Pulmonary Disease

Phenotypes of chronic obstructive pulmonary disease (COPD) and asthma may overlap in adults. However, these two conditions are immunologically and physiologically distinct. COPD is a chronic condition affecting large and small airways, as well as lung parenchyma and vasculature. Lung function is impaired over time, and airflow limitations are not fully reversible. The principal etiological factors linked to this condition are cigarette smoking and biomass exposure. Persistent pulmonary inflammation is amplified by gradual airway damage via oxidative stress and continuous release of proteases by various inflammatory cells. The major cause of morbidity and mortality is COPD exacerbations. Historically, bacterial pathogens have been considered a predominant etiological factor of infection in acute COPD exacerbations. In addition, acquisition of specific new pathogens, such as *Streptococcus pneumoniae* or *Moraxella catarrhalis*, as well as *P. aeruginosa*, contribute to increased rates of COPD exacerbations [58]. However, PCR-based techniques revealed the importance of different viruses in the exacerbated airway of COPD patients [17]. Likewise, a few studies demonstrated increased frequency and load of respiratory viruses in stable or mild COPD [59]. Viral colonization may be important in supporting an elevated inflammatory background. In addition, an increased number of macrophages and neutrophils in the small airways was found in stable COPD patients. An elevated inflammatory cell count together with increased total viral load was demonstrated to have an inverse relationship to the forced expiratory volume in 1 s/forced vital capacity ratio (FEV1/FVC). In addition, the use of glucocorticoids does favor viral persistence [60]. Latent adenoviral infection was suggested to be involved in the pathogenesis of COPD. Adenoviral detection was shown to be similar in stable or exacerbated COPD patients [7]. Additionally, a correlation was found between the lung destruction level and amount of adenovirus protein expression within alveolar epithelial cells [61]. Human bocavirus was identified in stable and exacerbated COPD patients, which supports the hypothesis that hBoV is either carried at low copy numbers or reactivated via another respiratory infection [62]. Viral–bacterial interactions are highly significant in COPD pathogenesis. Airway microbiomes in COPD patients are influenced by community-acquired respiratory viruses. Not to mention, COPD patients are more susceptible to community-acquired virus-induced airway infections that play a role in acute exacerbations of COPD. As shown with the influenza virus, viral inhibition of different antibacterial pathways may form a basis for secondary bacterial infection and worsen the outcome in COPD cases [60]. Challenges with RV altered the airway microbiome and triggered *Haemophilus influenzae* overgrowth in COPD patients [63].

## 7. Idiopathic Pulmonary Fibrosis

Idiopathic pulmonary fibrosis (IPF) does show dysregulated ecological balance within the respiratory microbiome. IPF is a rare interstitial lung disease. The specific characteristics of this disease are a gradual accumulation of extracellular matrix with worsening dyspnea, leading to impaired gas exchange. Pro-inflammatory conditions within the lung, exposure to medication, and immunodeficiency may contribute to viral reactivation. Of note, most of the exacerbations of IPF have been linked to noninfectious factors. Recently, it was postulated that occult infections may be important in IPF exacerbations. Different viral pathogens do contribute to the progression and aggravation of the disease. TTV was identified and studied in IPF patients and was detected at a similar frequency in IPF and acute lung injury cases [64]. This, however, does not contradict the potential role of TTV in the pathogenesis of IPF exacerbations. Different studies showed that the DNA or proteins of herpesvirus family members are increased in IPF patients. Anti-EBV IgA was identified in 60% of IPF blood samples. Meanwhile, 40% of IPF lung samples were positive for Epstein–Barr virus DNA. Cytomegalovirus (CMV) IgG was found in 80% of IPF patients [34]. Another study demonstrated the presence of Cytomegalovirus RNA in IPF airways. EBV, CMV, HHV7, or HHV8 were identified in 97% of all IPF patients by Tang et al. [65]. In addition, few groups have studied herpesvirus infection and its contribution to acute exacerbations of IPF. They uncovered an epithelial damage mechanism, which could be linked to a viral–mediated pathogenesis of acute exacerbations [34]. Likewise, a latent viral infection can reprogram epithelial and mesenchymal cells and may drive fibrotic processes over time [34]. As described before, the viral load within the respiratory tract of these patients is also a crucial factor for disease aggravation.

## 8. What Is Next?

Sensitive molecular methods have enabled the discovery of novel components of the airway virome. This constant evolution of genome sequencing can be a very powerful tool in explaining and analyzing the pathogenic effect of different viruses. Several questions are still unanswered, such as how much sampling of the human population is needed to perceive each detail of the respiratory virome to complete a “viral puzzle”. Additionally, certain DNA viruses have been discovered in stable chronic airway conditions, but their role in pathogenicity is still unclear. It is necessary to answer critical questions as to what a “healthy” lung virome is and which newly discovered viruses are pathogenic. The most important clinical question is, how could we preserve a stable airway condition? Moreover, how could we prevent and control acute exacerbations of chronic airway diseases?

The health–disease balance depends on various factors, but host immunity and specific characteristics of the microorganism (e.g., virulence, particle load, and other microorganisms) are the most significant in terms of a healthy lung and stable chronic conditions or acute exacerbations. As previously discussed, different resident viruses, bacteriophages, and their influence on a bacterial host, as well as transitory RNA viral infections are more often associated with impaired lung function and exacerbated conditions during chronic lung diseases. Therefore, it might be important to focus on specific antiviral treatment options and possible antiviral immunity regulation to keep airway conditions stable or improve inflammatory processes during exacerbations.

Analysis of interactions within the respiratory tract of the host and between the species could provide new insights into both the transcriptional and metabolic pathways. Changes in physiological processes of the local microbial communities manifest through metabolic turnover (e.g., anaerobic fermentation), which may be used as an early biomarker in predicting exacerbations of conditions [66]. Importantly, it could be used as a tool in monitoring response to different medications (e.g., antibiotics or glucocorticoids) or novel therapeutic methods. Additionally, microbiome profiling could be used as an indicator in characterizing chronic airway disease stability or progression. Microbe-derived biomarkers could predict pulmonary exacerbations and allow early interventions, even before airway inflammation is present. Alterations in viral–bacterial composition, ratio, and density tested in BALF could be used to assess disease progression or control labelling. This way, microbiome-derived metabolites could serve as new therapeutic targets in respiratory medicine.

A highly important aspect in restoring equilibrium in respiratory tract microbial communities could be bacteriophage application. With the increase of multidrug-resistant *P. aeruginosa*, phage-based agents could be employed as antimicrobial therapies [67,68]. Newly discovered novel phages within the CF-affected lung are of high interest due to their ability to control biomass in the airway. Human airways are unique in terms of drug delivery. Topical application via inhalation alone or together with systemic injection or a per os strategy, as well as direct targeting of the inflammatory zone could be used as possible routes for phage administration. Phages can reach the most distant lung parts and penetrate the tissue and the necessary phage concentrations could be reached within the vicinity of an ongoing inflammatory process. However, in a diseased lung, it could be more challenging compared to a healthy one, due to elevated numbers of inflammatory cells, protein-rich edema, etc. By contrast, an impaired lung epithelial–endothelial barrier with increased tissue permeability could benefit phage penetration from the systemic blood flow [69]. The best way of administration, as well as phage particle count, is still debatable. Another question is the efficacy of bacteriophage therapy. One of the most critical hallmarks of efficacy could be its effectiveness against *P. aeruginosa* biofilm. Biofilms are highly tolerant to various antimicrobials. In addition, antibiotic-resistant planktonic bacterial subpopulations are found within the biofilm. Antibiotics alone have been shown to be beneficial, mainly for planktonic bacteria. Temperate phages do modulate bacterial virulence factors and could inhibit bacterial biofilm formation [70]. They may weaken or destroy the biofilm matrix and favor the penetration of other agents (e.g., antibiotics) within it. This synergistic effect was proven in several in vitro studies [71]. Furthermore, lytic phages do not only infect bacteria but also do produce bacteria-directed lytic enzymes functionally similar to lysozymes, which can induce bacterial death. Both the transmembrane protein holin and a peptidoglycan cell wall hydrolase endolysin can trigger bacterial lysis [72]. For this reason, the engineering of recombinant proteins may be superior to simple phage administration. Phage-derived lytic enzyme action could be potentiated with additional antibiotic use. Additionally, lytic enzymes can favor antibiotic penetration into a bacterial biofilm. These characteristics make bacteriophage-based agents a highly attractive biotreatment method for chronic diseases, such as CF, COPD, or IPF [60]. Nevertheless, possible bacterial resistance to bacteriophages could be a considerable limitation in bacteriophage-derived therapy.

Various respiratory viruses, such as the influenza virus or RSV, are associated with acute exacerbations. These pathogens are transitory flora and may cause mild disease in previously healthy individuals. However, patients with chronic respiratory disease, such as asthma, CF, or IPF, are shown to suffer severe exacerbations caused by these RNA viruses. Yearly influenza immunization is highly indicated for these patients [36]. Additionally, Rhinovirus vaccines have been studied in in vivo animal models, to date, merely applicable in humans [73]. Respiratory syncytial virus vaccination was demonstrated to be effective among children and elderly adults and might decrease RSV infection in 75% of the cases [74].

A significant improvement has been made in viral–host pathogenetic mechanisms. Different treatment modalities to enhance the innate antiviral immune response have been studied as feasible therapeutic candidates in chronic respiratory conditions. Type I IFNs are the most important antiviral signaling pathway proteins in influenza virus- or RSV-induced respiratory conditions [75]. Type I IFN therapies have been extensively investigated. They were concluded, however, to not be effective because of their cost and side effects [75]. Toll-like receptor (TLR) 7/8 agonists may be beneficial in limiting the viral spread and preventing or reducing virus-induced acute exacerbations in chronic lung disease patients [73]. Likewise, inhibition of virus infection via small molecules or virus-specific antibodies has been researched for years. Palivizumab and motavizumab have demonstrated a beneficial effect against RSV. Motavizumab, however, needs larger studies to assess its role and safety in acute RSV infection. Different antivirals have been widely studied as treatment options. The only pharmacologically treatable respiratory virus is IV. Early detection and treatment with oseltamivir, a neuraminidase inhibitor, is rational to improve the exacerbation process [36]. However, a Cochrane systematic review reported a lack of evidence with regard to the validated efficacy against IV infection in CF patients [32]. Ribavirin, an anti-RSV agent, is no longer recommended; however, a few other anti-RSV options have emerged, such as immunoglobulins and siRNAs. Some antimicrobial agents, such as macrolides (e.g., azithromycin), demonstrated antiviral properties in vitro [76]. Clarithromycin and levocetirizine showed inhibitory effects on RV-induced bacterial adhesion to alveolar epithelial cells [77,78]. Several studies using antiviral medication against DNA viruses in different chronic lung diseases are very intriguing [34]. Therefore, special antiviral agents, antiherpes medication, for instance, may be of value in chronic disease control. Nonetheless, the use of such therapy in asymptomatic patients would be highly debatable. More scientific studies are needed to enhance knowledge regarding the persistence of the airway virome and its role in symptom-free chronic respiratory tract disease patients.

## 9. Conclusions

Growing data show a significant role of the airway virome in chronic respiratory disease exacerbations. With novel genome sequencing methods, more viruses were identified in healthy human lungs or chronic lung conditions. Number of studies have clarified the importance of the lung virome in shaping human immunity or the airway inflammation process. Recently, more bacteriophages were identified in airway samples. This part of the virome participates in the local airway inflammation process and immunity through infecting its direct host—a bacterial pathogen. Bacteriophages shape the local microbial ecosystem via eliminating its host, transferring antibiotic resistance genes, or adapting its host to the local environment of the respiratory tract. However, various phages have accounted for the largest part of the viral ecosystem in healthy lungs. Respiratory viruses, such as RV or RSV, have been shown to play a crucial role in pathogenesis of bronchiolitis or recurrent wheezing, leading to asthma development. These viruses, together with the influenza virus, parainfluenza virus, and human metapneumovirus, have been identified as significant pathogens in acute exacerbations of asthma, COPD, CF, or IPF. Moreover, airway samples from patients with exacerbated lung disease tested positive for herpes family viruses, such as CMV or EBV, or Anelloviruses, such as TTV, which were previously detected only in blood samples. These new data can already answer many questions related to airway disease exacerbation or asymptomatic inflammation. However, more studies are needed regarding the molecular mechanisms responsible for virus-induced exacerbations or viral persistence. A number of newly identified viruses may be further included in the screening and analysis process in chronic airway conditions and could clarify exacerbations when the pathogen is lacking. Their identification in healthy subjects could bring more light in understanding host–virus interactions. Moreover, new therapeutic methods could be investigated and may be implemented to prevent acute exacerbation or ameliorate a persistent inflammatory process. To date, bacteriophage therapy, some antivirals, or metabolic modulation of microbial products seem to be futuristic treatment approaches. However, prevention tactics, such as seasonal vaccination or neuraminidase inhibitors during infection with the influenza virus, have shown to be beneficial in acute exacerbations of chronic respiratory diseases. Nevertheless, more antiviral strategies must be tested and implemented. Furthermore, it is still unclear if the presence of viral agents during the stable asymptomatic chronic airway condition should be treated. Thus, it remains to be discussed if different viral pathogens can represent specific disease phenotypes or predict their exacerbations or outcomes. Nevertheless, a better understanding of the of viruses in different chronic lung diseases may lead to better control over inflammatory processes in the airways and a prospect in reaching more efficient therapy for chronic respiratory conditions.
